# Serum Spike Protein Persistence Post COVID Is Not Associated with ME/CFS

**DOI:** 10.3390/jcm14041086

**Published:** 2025-02-08

**Authors:** Annick Fehrer, Franziska Sotzny, Laura Kim, Claudia Kedor, Helma Freitag, Cornelia Heindrich, Patricia Grabowski, Nina Babel, Carmen Scheibenbogen, Kirsten Wittke

**Affiliations:** 1 Institute of Medical Immunology, Charité–Universitätsmedizin Berlin, Corporate Member of Freie Universität Berlin and Humboldt Universität zu Berlin, Augustenburger Platz 1, 13353 Berlin, Germany; 2 Center for Regenerative Therapies (BCRT), Berlin Institute of Health (BIH), Charité-Universitätsmedizin Berlin, Charitéplatz 1, 10117 Berlin, Germany; 3 Center for Translational Medicine and Immune Diagnostics Laboratory, University Hospital of the Ruhr-University Bochum, Hölkeskampring 40, 44625 Herne, Germany

**Keywords:** COVID-19, chronic fatigue syndrome, ME/CFS, long COVID, post-COVID syndrome, spike protein, SARS-CoV-2, viral persistence

## Abstract

**Background/Objectives**: According to the World Health Organization (WHO) and Centers for Disease Control and Prevention (CDC), an estimated 3–6% of people suffer from post-COVID condition or syndrome (PCS). A subset meets the diagnostic criteria for myalgic encephalomyelitis/chronic fatigue syndrome (ME/CFS). Studies have reported that SARS-CoV-2 proteins or RNA can persist after acute infection in serum or tissues, but their role in PCS is unclear. **Methods**: Here, SARS-CoV-2 spike protein was analyzed in the serum of 121 PCS patients with predominant fatigue and exertional intolerance, of whom 72 met diagnostic criteria for ME/CFS, 37 post-COVID recovered healthy controls, and 32 pre-pandemic healthy controls. **Results**: Spike protein was detected in the serum of 11% of recovered controls, 2% of PCS patients, and 14% of ME/CFS patients between 4 and 31 months after SARS-CoV-2 infection, but not in pre-pandemic samples. The occurrence and concentration of spike protein did not correlate with infection or vaccination timepoints. In ME/CFS patients, spike protein presence was not associated with the severity of symptoms or functional disability. In 5 out of 22 patients who under-went immunoglobulin depletion, spike protein levels were reduced or undetectable after treatment, indicating binding to immunoglobulins. **Conclusions**: In summary, this study identified serum spike protein in a subset of patients but found no association with ME/CFS.

## 1. Introduction

Post-COVID syndrome (PCS) refers to persistent symptoms that occur within three months of an infection with severe acute respiratory syndrome coronavirus type 2 (SARS-CoV-2) and last for more than two months, according to the World Health Organization (WHO) definition [1]. It affects individuals across a broad demographic spectrum but is particularly prevalent among young and middle-aged women. PCS is a major burden for health care, economics, and society with an estimated 3–6% of patients suffering from chronic and often debilitating illness and no causal therapy so far [1,2,3,4].

PCS is multisystemic, affects various bodily functions, and presents with a variety of symptoms that include fatigue, exercise intolerance with post-exertional malaise (PEM), cognitive impairments, headaches, muscle pain, and autonomic dysfunction among others. The clinical trajectory can be persistent, relapsing, or fluctuating, significantly impairing the quality of life and work capacity for many affected individuals. A subset of PCS patients meets the Canadian consensus (CCC) or Institute of Medicine (IOM) criteria for the diagnosis of myalgic encephalomyelitis/chronic fatigue syndrome (ME/CFS) [5,6,7].

Although the exact pathomechanisms remain unclear, there are numerous studies providing evidence for inflammation, antibody-mediated autoimmunity, vascular inflammation and circulatory dysfunction, Epstein-Barr virus (EBV) reactivation, as well as SARS-CoV-2 persistence in PCS [8,9].

While in the majority of individuals infected with SARS-CoV-2 the virus is successfully cleared from the respiratory tract and blood within one to two weeks in correspondence with the resolution of clinical symptoms, a subset of patients experience a prolonged viral persistence or reactivation [10]. In these patients, recurrent episodes of viral shedding, as well as persisting viral RNA or spike antigen may contribute to continued activation of immune responses, possibly inducing persistent tissue inflammation [10,11]. Contrary to the common perception of coronavirus disease 2019 (COVID-19) as a respiratory disease, SARS-CoV-2 cell tropism was not only identified in lungs and trachea but also in the kidneys, pancreas, brain, heart, skin, blood vessels, and small intestines [12]. Apart from primary infection of the respiratory epithelium, other cell types like gastrointestinal epithelia, endothelial cells, and neurons may also be susceptible to infection [10]. SARS-CoV-2 RNA was detected in non-respiratory tissues, including the brain, up to several months after infection [13,14]. Several studies have reported persistent SARS-CoV-2 viral reservoirs or antigens, such as spike protein, in some patients for up to two years, with prevalence rates up to 60% in PCS patients compared to much lower rates in recovered controls [15,16,17,18,19,20,21]. However, these studies often lack comprehensive patient characterization, correlation with clinical data, and adequate controls to validate findings [17,18,19,20,21,22,23].

To this date, the relationship between PCS and viral persistence is not resolved. Here, spike protein in the serum of PCS patients, as well as pre-pandemic and recovered healthy controls, was quantified, and a potential correlation of viral persistence with clinical manifestation and symptom severity was analyzed. In addition, the effect of immunoglobulin depletion via immunoadsorption (IA) on serum spike protein was investigated [24]. The purpose of this study was to further clarify the role of viral persistence in the pathogenesis of PCS and ME/CFS and to explore its potential implication as a diagnostic marker and for treatment strategies.

## 2. Materials and Methods

### 2.1. Study Design, Study Cohorts, and Symptom Assessment

An exploratory, cross-sectional study investigating serum spike persistence in individuals with and without PCS symptoms up to 38 months after SARS-CoV-2 infection was conducted. This study aimed to determine whether serum spike protein is associated with PCS symptoms or correlates with disease severity and selected laboratory markers.

Serum samples and clinical data from 121 PCS patients suffering from moderate-to-severe fatigue and exertion intolerance following mostly mild COVID-19 were collected between October 2020 and January 2024 at the ME/CFS outpatient clinic of the Institute of Medical Immunology, Charité, Berlin. From these, 72 met the CCC for diagnosis of ME/CFS [25,26]. Relevant cardiac, respiratory, neurological, or psychiatric comorbidities were excluded. Furthermore, 22 ME/CFS patients who had participated in an observational study of immunoglobulin depletion via IA (IA-ME/CFS) were analyzed [24]. Of these, serum samples at timepoints before and four weeks after IA were analyzed.

Additionally, serum samples from 37 post-COVID recovered healthy controls (pcHCs) five to twelve months after their last SARS-CoV-2 infection were obtained between January 2023 and October 2023. pcHCs did not suffer from a disease with relevant impairment or take regular medication and had no infection or vaccination within the last four weeks. To control for assay specificity, 32 pre-pandemic serum samples from pre-pandemic healthy controls (ppHCs), collected between January 2019 and October 2019, were analyzed. Cohort characteristics are listed in Table 1. The IA-ME/CFS study group differed significantly from the other study groups due to a longer disease duration, as well as longer time after SARS-CoV-2 infection and vaccination. Severity of disease and symptoms were quantified using questionnaires. PEM was assessed according to Cotler et al. 2018 with scores ranging from 0 to 46 (no to frequent, severe, long lasting PEM) [27]. Functional ability was scored using the Bell Disability Scale ranging from 0 to 100 (total loss of self-dependence to no restrictions) [28]. Daily physical activities were assessed using the Short Form Health Survey 36 (SF-36) ranging from 0 to 100 (greatest to no restrictions) [29]. The severity of the cardinal symptoms of fatigue, cognitive impairment, immune symptoms, and pain were quantified using a Likert scale (1 = no symptoms to 10 = most severe symptoms). Symptoms of autonomic dysfunction were assessed using the Composite Autonomic Symptom Score 31 (COMPASS-31) ranging from 0 to 100 (no to strongest symptoms) [30]. As evident from the Bell score, ME/CFS patients suffered from more severe disease compared to PCS non-ME/CFS patients, while the clinical presentation of ME/CFS and IA-ME/CFS study groups were comparable (Table 1). Ethical Committee approval for this project was obtained in accordance with the 1964 Declaration of Helsinki and its later amendments (EA2/066/20 and EA2/067/20). All study participants signed informed consent before study inclusion.

### 2.2. Quantification of Serum Spike RBD and Anti-S1 IgG

Whole blood samples from participants were allowed to clot at room temperature for at least 30 min and then centrifuged at 2000× *g* for 10 min at room temperature. Serum was collected and stored at −80 °C. Serum concentration of the SARS-CoV-2 spike receptor-binding domain (RBD) was determined using a Human SARS-CoV-2 RBD ELISA Kit purchased from Thermo Fisher Scientific (EH492RB, Schwerte, Germany) and conducted according to the manufacturer’s instructions. Serum anti-S1 immunoglobulin G (IgG) was quantified using an anti-SARS-CoV-2 QuantiVac ELISA (IgG) purchased from Euroimmun (EI 2606-9601-10 G, Lübeck, Germany) and was conducted according to the manufacturer’s instructions. Obtained arbitrary units (AUs) were converted into the WHO international standard for anti-SARS-CoV-2 immunoglobulin binding activity (Binding Antibody Units, BAUs) by multiplying by a factor of 3.2.

### 2.3. Assessment of Routine Laboratory and Functional Parameters

D-Dimer, platelet count, mean platelet volume (MPV), and interleukin-8 (IL-8) (post erythrocyte lysis) were determined at the Charité diagnostics laboratory Labor Berlin GmbH (Berlin, Germany). Levels of autoantibodies (AABs) against the G protein-coupled receptor (GPCR) β2 adrenergic receptor (β2-AdR) were determined by CellTrend GmbH (Luckenwalde, Germany) using indirect ELISA technology.

Peripheral endothelial function was assessed in the IA-ME/CFS study group by the reactive hyperemia index (RHI) using postocclusive reactive hyperemia peripheral arterial tonometry (RH-PAT) (endoPAT2000, Itamar Medical Ltd., Caesarea, Israel) as previously described [31].

### 2.4. Data Collection and Management

Study data, including clinical and routine laboratory parameters, were collected and managed using REDCap electronic data capture tools hosted at Charité—Universitätsmedizin Berlin [32,33].

### 2.5. Statistical Analysis

Study data are presented as the median with the range or interquartile range (IQR) for each study group (*n* = number of individuals in the study group) as indicated. Nonparametric statistical methods were used. Univariate comparisons of two independent groups were conducted using the Mann–Whitney U test and for multiple independent groups using the Kruskal–Wallis test with Benjamini–Hochberg correction of multiple comparisons. The distribution of categorical data was compared using the chi-square test. Correlation was assessed using Spearman’s rank-order correlation. A two-tailed *p* value < 0.05 was considered significant. Microsoft Excel 2016 was used for data analysis, and GraphPad Prism 9 was used for statistical analysis and graphical presentation.

## 3. Results

### 3.1. Spike Protein Is Detectable in Serum of Individuals After Acute SARS-CoV-2 Infection

**Figure 1:**
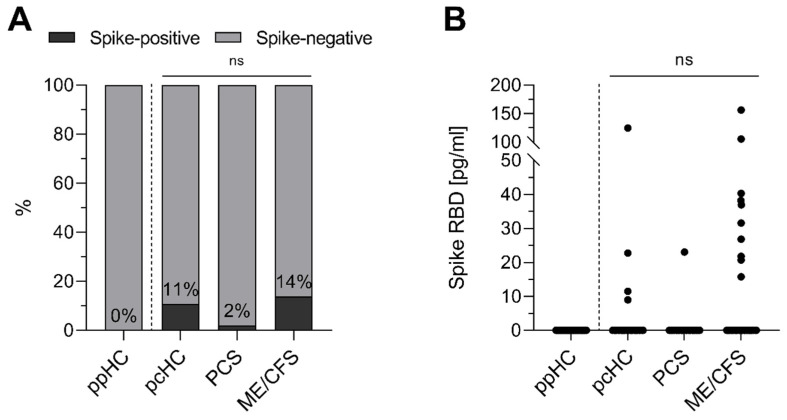
The serum spike RBD concentration of pre-pandemic healthy controls (ppHCs), post COVID healthy controls (pcHCs), post-COVID syndrome (PCS) patients, and post-COVID ME/CFS patients. Spike RBD was quantified in the serum of ppHC (*n* = 32), pcHC (*n* = 37), PCS (*n* = 49), and ME/CFS (*n* = 72) patients. (**A**) The frequency of spike RBD positive (black) and spike RBD negative (grey) individuals in the different study cohorts. Percentages inside the bars indicate the frequency of spike-positive individuals. (**B**) The individual serum spike RBD concentration [pg/mL] of study cohorts. ns = not significant.

SARS-CoV-2 spike protein was quantified by ELISA detecting RBD in the serum of three post-COVID cohorts: 37 pcHCs and 121 PCS patients with predominant fatigue and exertional intolerance, of whom 72 met ME/CFS diagnostic criteria, 1 to 38 months after their last SARS-CoV-2 infection. Additionally, a cohort of 32 ppHCs was analyzed to validate assay specificity (Figure 1). While all ppHC serum samples tested negative for SARS-CoV-2 spike protein, 11% of the pcHC serum samples, 2% of PCS non-ME/CFS serum samples, and 14% of the ME/CFS serum samples were positive 4–31 months after infection (Figure 1A). Both frequency and spike protein concentration (Figure 1B) did not differ significantly between the three post-COVID cohorts. However, when comparing only the patient cohorts, there was a significant difference in the prevalence of spike protein in serum (*p* = 0.0261).

### 3.2. Serum Spike Protein Concentration Is Independent of the Time Since Last SARS-CoV-2 Spike Protein Contact

**Figure 2:**
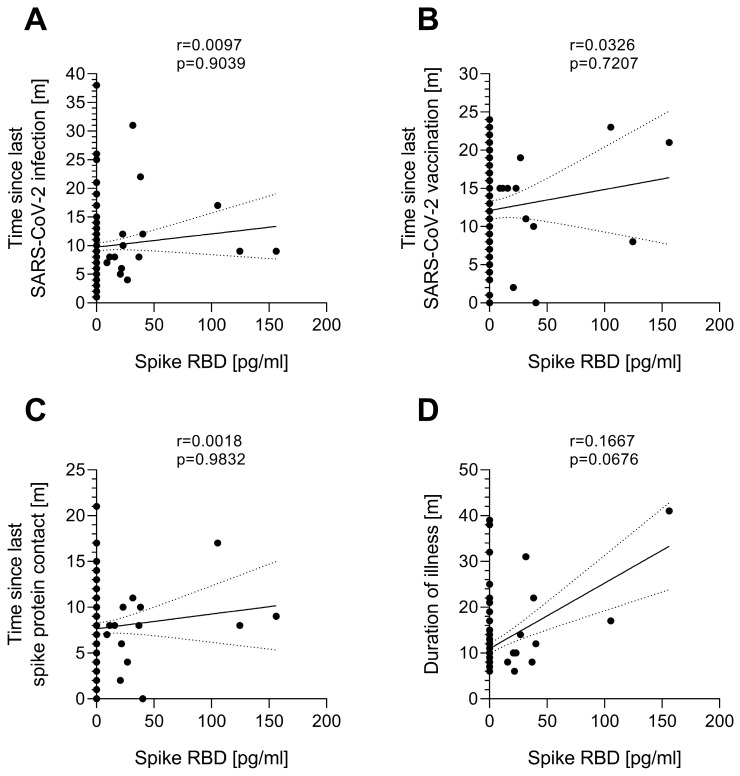
The correlation analysis of spike RBD in the serum of post-COVID study groups with the time since SARS-CoV-2 infection or vaccination. The spike RBD concentration in serum of post-COVID healthy controls (pcHCs, *n* = 37), post-COVID syndrome (PCS) patients (*n* = 49), and post-COVID ME/CFS patients (*n* = 72) was correlated with (**A**) the time since last SARS-CoV-2 infection, (**B**) the time since the last SARS-CoV-2 vaccination, (**C**) the time since the last contact with spike protein either during SARS-CoV-2 infection or vaccination, and (**D**) the duration of illness for patients. Correlation was assessed using Spearman’s rank-order correlation. Graphs are fitted with a simple linear regression line (black line) and 95% confidence intervals (dotted black lines). Correlation coefficient (r) and significance level (*p*) are displayed at the top of each graph.

Next, a potential relationship between serum SARS-CoV-2 spike protein and time since last infection or vaccination in all three post-COVID study groups (pcHC, PCS, and ME/CFS) was investigated. Neither the time since the last SARS-CoV-2 infection (Figure 2A), time since last SARS-CoV-2 vaccination (Figure 2B), nor time since last spike protein contact by either infection or vaccination (Figure 2C) correlated with the serum spike protein presence or concentration. Furthermore, there was no correlation between the time since the illness-triggering SARS-CoV-2 infection and serum spike protein concentration in PCS or ME/CFS patients (Figure 2D). Two out of ten spike-positive ME/CFS patients were vaccinated against SARS-CoV-2 shortly before their visit (3 and 13 weeks, respectively). Among the spike-negative individuals, five ME/CFS and four PCS patients were vaccinated within 6 weeks before their visit, and four ME/CFS and four PCS patients within 16 weeks before their visit. Two spike-negative patients had a SARS-CoV-2 infection seven or twelve weeks prior.

### 3.3. Serum Spike Protein Is Not Associated with ME/CFS Disease and Symptom Severity

Persistent viral reservoirs in PCS are presumed to be associated with inflammation and hypercoagulability, which may contribute to impaired blood flow, endothelial dysfunction, and PCS symptoms [8,10,34]. A potential association between serum spike protein and severity of disease and key symptoms was evaluated by comparing the 10 spike-positive and 62 spike-negative ME/CFS patients. No significant differences were observed in the severity of disability, assessed by Bell and SF-36 physical function scores, nor in the PEM score, severity of fatigue, pain, cognitive, immune, or autonomic symptoms, as assessed by the COMPASS-31, between spike-positive and spike-negative ME/CFS patients (Table 2). The 49 PCS patients not fulfilling ME/CFS criteria were not analyzed as there was only 1 spike-positive patient in this study group.

A subset of ME/CFS patients had elevated IL-8 and a few had elevated D-dimer levels, platelet count, or MPV as potential markers of endothelial inflammation or hypercoagulation. Levels of these markers were not associated with serum spike persistence in this study cohort (Table 3A). Patients who were treated within the IA study had elevated levels of β2 adrenergic receptor autoantibodies (β2-AdR-AABs). No significant differences in levels of β2-AdR-AABs between spike-positive and spike-negative ME/CFS, nor in levels of anti-S1-IgG were found (Table 3B). Moreover, 6 of the 21 ME/CFS patients showed evidence for endothelial dysfunction as assessed by the RHI using postocclusive RH-PAT, but again, no association with spike persistence was observed (Table 3C).

### 3.4. Serum Spike Protein Is Reduced by Immunoadsorption (IA) to Deplete Immunoglobulins

**Figure 3:**
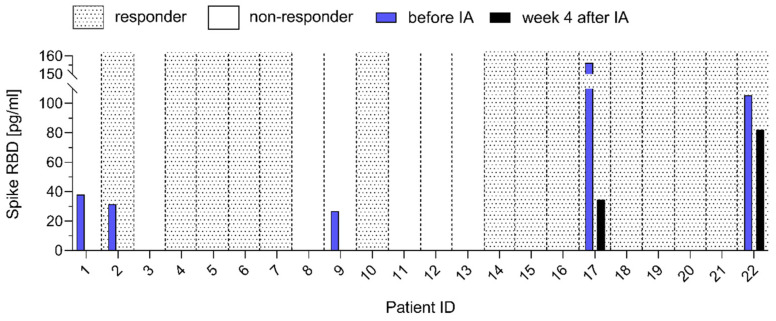
Spike RBD concentration in serum of post-COVID ME/CFS patients before immunoadsorption (IA) (IA-ME/CFS) and four weeks after IA. Patients who responded to IA are indicated by dotted black background and those who did not are indicated by a white background based on the improvement in SF-36 physical function four weeks after therapy [24]. Spike RBD concentration [pg/mL] in patients’ serum was determined before (blue bar) and four weeks after IA (black bar).

Twenty-two of the ME/CFS patients studied here were treated with IA for immunoglobulin depletion (IA-ME/CFS) as reported elsewhere [24]. Levels of SARS-CoV-2 spike protein were measured before and four weeks after IA (Figure 3). Interestingly, in all five patients with detectable serum spike protein, levels were reduced in two and no longer detectable in three patients four weeks after IA. Patient #17 had a SARS-CoV-2 infection immediately before the four-weeks-after-IA visit. Three of the five spike-positive patients treated with IA were responders, and two were non-responders as defined by improvement in their physical function (SF-36) four weeks after IA [24]. Thus, no evidence for an association between spike protein serum persistence and clinical response to immunoglobulin depletion was found.

## 4. Discussion

The present study shows spike protein in serum up to 31 months after infection in a subset of PCS patients with fatigue and exertional intolerance, as well as in asymptomatic individuals. Neither an association with PCS symptoms nor with the severity of symptoms in the ME/CFS subgroup was found. Moreover, no link to markers of endothelial dysfunction, inflammation, or hypercoagulation was found.

The finding from this and other studies of the absence of viral proteins in pre-pandemic samples provides strong evidence for the specificity of the detection of spike protein after SARS-CoV-2 infection in a subset of individuals. The prevalence of spike persistence in serum in the present study is consistent with other studies [18,35]. Peluso et al. reported persistence of SARS-CoV-2 antigen, most commonly the spike protein, in 9.2% of 660 pandemic-era plasma samples up to 14 months after SARS-CoV-2 infection [18]. A recent large study by Swank et al., analyzing four different cohorts, found spike protein in the serum of 0–5% of asymptomatic individuals and 2–12% of the ME/CFS subgroup up to 14 months after infection. Higher frequencies, with 10–26% spike-positive samples, were found in all four cohorts within the subgroups of patients with cardiopulmonary symptoms [35]. In the well-characterized ME/CFS cohort analyzed in our study, spike protein levels and symptom severity were examined for potential correlation, but no association was found.

Others reported persistent spike protein or viral RNA in the blood of PCS patients for up to twelve months after infection with a prevalence of up to 60%, while none was detected in 26 recovered controls in one study [19] or was detected less frequently in recovered controls in other studies [20,36]. Haddad et al. recently reported an ongoing immune response against SARS-CoV-2 antigens evident from newly activated antibody-secreting cells, suggesting viral persistence or reactivation in 40% of 61 analyzed PCS patients and none of the 25 analyzed recovered controls that were studied [21]. However, these studies did not explicitly match the time points of SARS-CoV-2 infection or vaccination of their study groups and did not document potential reinfections in the time frame investigated. In addition, studies often lack comprehensive patient characterization and classification as well as correlation with clinical data, or they do not include sufficient controls to contextualize results and verify assay specificity [17,18,19,20,21,22,23]. In contrast, one study was unable to detect any viral RNA by transcriptome analysis in the blood of either PCS patients or controls [37]. The heterogeneity of findings in the various studies may further be related to the generally low levels of viral proteins and the different tests that were used.

We found spike protein in serum to be independent of the time since the last SARS-CoV-2 infection or vaccination. This suggests that spike protein presence is independent of a recent exposure, but rather depends on individual differences in viral clearance, or persisting viral reservoirs, which needs to be clarified by further investigation.

Spike protein can promote microvascular inflammation and thrombogenic processes [38,39], but the present study did not find a correlation between endothelial dysfunction, thromboinflammatory markers, and spike protein persistence in ME/CFS patients.

The hypothesis of viral persistence playing a role in the pathomechanism of PCS is currently used as a rationale for antiviral treatment studies. Paxlovid, a drug consisting of the antiviral agents nirmatrelvir and ritonavir, has been shown to be effective in preventing a severe course of COVID-19 [40], which is associated with an increased risk of PCS [41,42]. Findings from a study using a large dataset of >281,000 participants from the US Department of Veterans Affairs suggested a protective effect of Paxlovid treatment during acute COVID-19 also for the development of PCS [43], while other smaller studies could not reproduce these findings [44,45]. A first randomized placebo-controlled trial of 155 PCS patients in the STOP-PASC study showed no significant effect of 15 days of Paxlovid treatment on persistent symptoms [46]. However, in this study, viral particles were not assessed; thus, it is not possible to draw conclusions about the subgroup of patients with evidence of viral persistence.

Further, ME/CFS patients treated with IA for immunoglobulin depletion were studied, and a reduction in or removal of spike protein was observed. This suggests that spike protein is bound to IgG. However, it cannot be excluded that there is an unspecific binding of spike protein to the antibody-specific sepharose gel matrix used for the IA procedure. The presence of or reduction in serum spike protein was not associated with treatment response. This further suggests that persistent spike protein does not cause symptoms in ME/CFS patients.

The present study has some limitations that need to be addressed. It is a cross-sectional study that provides a snapshot of serum spike persistence. Temporal profiling of SARS-CoV-2 antigens in the plasma of PCS patients showed fluctuating concentration profiles of both spike and nucleocapsid protein over a time period of up to twelve months after infection [19]. Here, levels of S1 spike protein were analyzed by the detection of RBD. This is worth mentioning, as other studies reported discrepancies between S1 and full spike protein as well as nucleoprotein levels in serum [19]. In addition, spike protein may not only be present in a soluble form but may also be transported in extracellular vesicles, as previously reported [20,47]. Furthermore, the individuals investigated in this study may have been infected with different variants of SARS-CoV-2, which has not been analyzed and may affect spike persistence, too.

Other studies have shown persistence of viral RNA in tissues [13,14,15,16,17]. The present study focused on a PCS subgroup with predominant fatigue and exertional intolerance. As PCS is a highly heterogeneous disease, viral persistence may play a role only in certain subtypes of PCS as found by Swank et al. [35]. Further research is needed to elucidate potential differences in spike persistence in clinically and immunologically distinct PCS subtypes and its potential relevance for the diagnosis and treatment of patients.

## 5. Conclusions

In conclusion, despite the limitations described, this study provides further evidence for serum spike persistence in a subset of individuals after SARS-CoV-2 infection but found no association with ME/CFS or symptom severity.

## Figures and Tables

**Table 1:** Cohort characteristics.

Study Group	ppHC	pcHC	PCS	ME/CFS	IA-ME/CFS	*p*-Values
*n*	32	37	49	50	22	NA
Age [y], median (range)	29 (21–54)	34 (23–56)	37 (23–62)	40.5 (21–59)	40 (31–59)	*p*1 = 0.2463 *p*2 = 0.1484 *p*3 = 0.0824 *p*4 = 0.6535 *p*5 = 0.3027 *p*6 = 0.4243
Female sex [%]	77.14	78.38	79.59	80.00	68.18	*p* = 0.8230
Duration of illness [m], median (range)	NA	NA	9 (6–14)	9 (6–17)	20 (8–41)	*p*4 = 0.3443 ***p*5 < 0.0001** ***p*6 < 0.0001**
Time after last SARS-CoV-2 infection [m], median (range)	NA	10 (5–12)	8 (1–13)	9 (5–12)	14 (2–38)	*p*1 = 0.0679 *p*2 = 0.3034 *p*3 = 0.2233 *p*4 = 0.2819 ***p*5 = 0.0055** *p*6 = 0.0504
Time after last SARS-CoV-2 vaccination [m], median (range)	NA	16 (4–22) 0 unvacc. *n* = 24	9 (0–20) 9 unvacc. *n* = 47	11.5 (0–24) 6 unvacc. *n* = 49	17 (0–24) 1 unvacc. *n* = 20	***p*1 = 0.0032** *p*2 = 0.0955 *p*3 = 0.3700 *p*4 = 0.1049 ***p*5 = 0.0004** ***p*6 = 0.0133**
Bell Disability Scale, median (range)	NA	NA	50 (30–90)	30 (10–60)	30 (20–40)	***p*4 < 0.0001** ***p*5 < 0.0001** *p*6 = 0.4806

The Kruskall–Wallis rank-sum test or Mann–Whitney U rank-sum test were performed to test the statistical significance of the difference in numerical data between multiple or two of the post-COVID cohort groups (pcHC, PCS, and ME/CFS), respectively. The distribution of categorical data was compared using the chi-square test. *p*1 = PCS vs. pcHC, *p*2 = ME/CFS vs. pcHC, *p*3 = IA-ME/CFS vs. pcHC, *p*4 = PCS vs. ME/CFS, *p*5 = PCS vs. IA-ME/CFS, and *p*6 = ME/CFS vs. IA-ME/CFS. ppHC = pre-pandemic healthy control, pcHC = post-COVID healthy control, PCS = PCS non-ME/CFS, ME/CFS = post-COVID ME/CFS, IA-ME/CFS = Immunoadsorption ME/CFS (ME/CFS subcohort before IA), unvacc. = unvaccinated, y = years, m = months, and NA = not assessed. Significant differences are highlighted in bold.

**Table 2:** Comparative analysis of disease and symptom severity in ME/CFS patients with (Spike+) or without (Spike−) persistent serum spike RBD.

Parameter	Spike+ (*n* = 10) Median (Range) *n*	Spike− (*n* = 62) Median (Range) *n*	*p*-Value
Severity of disability and PEM			
Bell Disability Scale	30.00 (20.00–40.00) *n* = 10	30.00 (10.00–60.00) *n* = 61	0.7380
SF-36 Physical Functioning	25.00 (5.00–50.00) *n* = 10	35.00 (0.00–70.00) *n* = 61	0.1021
PEM score	36.00 (29.00–46.00) *n* = 9	34.00 (19.00–46.00) *n* = 58	0.5707
Symptom scores			
Fatigue score	8.25 (6.25–10.00) *n* = 9	8.00 (5.5–10.00) *n* = 59	0.8686
Cognitive score	6.67 (1.67–9.33) *n* = 9	7.00 (2.33–10.00) *n* = 60	0.9265
Immune score	2.67 (1.00–7.33) *n* = 8	3.67 (1.00–8.67) *n* = 58	0.2961
Headache	6.00 (1.00–10.00) *n* = 9	6.50 (1.00–10.00) *n* = 60	0.5623
Muscle pain	6.00 (1.00–10.00) *n* = 9	7.00 (1.00–10.00) *n* = 60	0.6602
Joint pain	6.00 (1.00–9.00) *n* = 9	5.00 (1.00–10.00) *n* = 60	0.8496
Assessment of autonomic dysfunction			
COMPASS-31 total	41.51 (11.55–58.24) *n* = 10	38.25 (2.68–76.23) *n* = 59	0.4045

The Mann–Whitney U rank-sum test was performed to test the statistical significance of the difference in numerical data between the study groups. SF-36 = Short Form Health Survey 36, PEM = post-exertional malaise, and COMPASS-31 = Composite Autonomic Symptom Score 31.

**Table 3:** Comparative analysis of routine laboratory and functional markers of ME/CFS patients with (Spike+) or without (Spike−) persistent serum spike RBD.

Parameter	Reference Range	Spike+ (*n* = 10) Median (IQR) *n* (out of Reference Range)/*n*	Spike− (*n* = 62) Median (IQR) *n* (out of Reference Range)/*n*	*p*-Value
**(A)**	**Thrombotic and Inflammatory Markers**			
IL-8 [pg/mL]	<150.00	126.80 (94.60–132.10) 1/7	131.00 (106.20–176.90) 18/55	0.2705
D-Dimer [mg/L]	<0.50	0.21 (0.19–0.34) 0/5	0.23 (0.19–0.38) 3/39	0.5863
Platelets [/nL]	150.00–370.00	258.50 (244.50–277.00) 0/8	265.50 (220.75–308.50) 3/60	0.9513
MPV [fL]	7.00–12.00	10.90 (10.45–11.55) 1/8	10.70 (10.20–11.60) 4/60	0.5273
**(B)**	**β2-AdR-AAB and Anti-S1 IgG**			
β2-AdR-AAB [a.u.]	<14.00	24.15 (10.2–34.79) 6/10	15.63 (8.51–24.50) 31/59	0.2613
Anti-S1 IgG [BAU/mL] median, (IQR), *n*	1953.51 (1034.04–3265.13) *n* = 16 *	838.33 (447.05–1957.15) *n* = 10	1185.01 (547.86–1944.94) *n* = 32	0.7382
**(C)**	**Markers of Endothelial Dysfunction**			
RHI	>1.67	1.78 (1.41–2.19) 2/4	2.08 (1.70–2.37) 4/17	0.4531

* Median (IQR) of 16 pcHCs. The Mann–Whitney U rank-sum test was performed to test the statistical significance of the difference in numerical data between the study groups. IQR = interquartile range, MPV = mean platelet volume, β2-AdR = β2 adrenergic receptor, AAB = autoantibody, IgG = immunoglobulin G, S1 = spike protein subunit 1, BAU = Binding Antibody Units, and RHI = reactive hyperemia index.

## Data Availability

The data presented in this study are available on request from the corresponding author.

## Abbreviations

The following abbreviations are used in this manuscript:

AAB	Autoantibody
AU	Arbitrary units
BAU	Binding Antibody Unit
β2-AdR	β2 adrenergic receptor
CCC	Canadian Consensus Criteria
CDC	Centers for Disease Control and Prevention
COMPASS-31	Composite Autonomic Symptom Score 31
COVID-19	Coronavirus disease 2019
EBV	Epstein-Barr virus
GPCR	G protein-coupled receptor
IA	Immunoadsorption
IL-8	Interleukin-8
IOM	Institute of Medicine
IgG	Immunoglobulin G
IQR	Interquartile range
m	Months
ME/CFS	Myalgic encephalomyelitis/chronic fatigue syndrome
MPV	Mean platelet volume
NA	Not assessed
ns	Not significant
PCS	Post-COVID syndrome
PEM	Post-exertional malaise
RBD	Receptor-binding domain
RH-PAT	Reactive hyperemia peripheral arterial tonometry
RHI	Reactive hyperemia index
SARS-CoV-2	Severe acute respiratory syndrome coronavirus type 2
SF-36	Short Form Health Survey 36
unvacc.	Unvaccinated
WHO	World Health Organization
y	Years
pcHC	Post-COVID healthy control
ppHC	Pre-pandemic healthy control
